# Short‐Term Change in IgG Antibody Elicited by Omicron BA.5 Infection and Inhaled Ad5‐nCoV Vaccine Among Healthcare Workers

**DOI:** 10.1002/iid3.70332

**Published:** 2026-01-19

**Authors:** Zhaohui Luo, Ting Zeng, Shi Zhao, Jing Liang, Shengzhi Sun, Yongkang Ni, Chunyan Yan, Liang Yin, Lan Wang, Kai Wang, Zihao Guo

**Affiliations:** ^1^ The Sixth Affiliated Hospital of Xinjiang Medical University Urumqi China; ^2^ School of Public Health Xinjiang Medical University Urumqi China; ^3^ School of Public Health Tianjin Medical University Tianjin China; ^4^ School of Public Health Capital Medical University Beijing China; ^5^ Department of Medical Engineering and Technology Xinjiang Medical University Urumqi China; ^6^ JC School of Public Health and Primary Care Chinese University of Hong Kong Hong Kong China

**Keywords:** antibodies, inhaled adenovirus type 5 vector vaccine, Omicron variants, SARS‐CoV‐2

## Abstract

**Background:**

Little is known about the immune response induced by the SARS‐CoV‐2 Omicron BA.5 variants or the inhaled adenovirus vector vaccine. In this study, we investigated the short‐term profile of antireceptor‐binding‐domain IgG antibody responses elicited by either the virus or the vaccine.

**Methods:**

A cohort of healthcare workers who were infected with Omicron BA.5 or who received the inhaled adenovirus vector vaccine was identified between August and December 2022. Blood samples were collected twice to detect IgG antibodies against the receptor‐binding domain of the SARS‐CoV‐2 spike protein. Baseline characteristics were obtained through questionnaires. Multivariate linear mixed‐effect models were applied to assess the changes in IgG antibody levels between the two laboratory test time points, adjusting for baseline covariates.

**Results:**

Among 1146 identified healthcare workers, a total of 419 healthcare workers with known infection status who provided informed consent were eligible for inclusion in the study. The study participants were mostly female (71.8%), had received three doses of intramuscularly injected inactivated vaccine before follow‐up (93.8%), and had no comorbidities (94.3%). We estimated that the IgG level increased by 5.1% per week over the 2 months following Omicron BA.5 infection. No significant change in IgG antibody levels in the short term was observed within 1 month after receiving the inhaled adenovirus vector vaccine.

**Conclusion:**

These findings suggested that the inhaled adenovirus vector vaccine may provide modest protection against Omicron BA.5 infection in the healthcare worker population.

## Graphical abstract

In this study, we recruited a cohort of healthcare workers who were infected with Omicron BA.5 or received inhaled adenovirus vector vaccine to investigate the short‐term profile of antireceptor‐binding‐domain IgG antibody elicited by the virus or vaccine. By using mixed‐effect models with potential confounders adjusted we estimated the IgG level increased by 5.1% per week within 2 months after Omicron BA.5 infection. No significant change in IgG antibody in short term was observed 1 month after receiving the inhaled adenovirus vector vaccine.

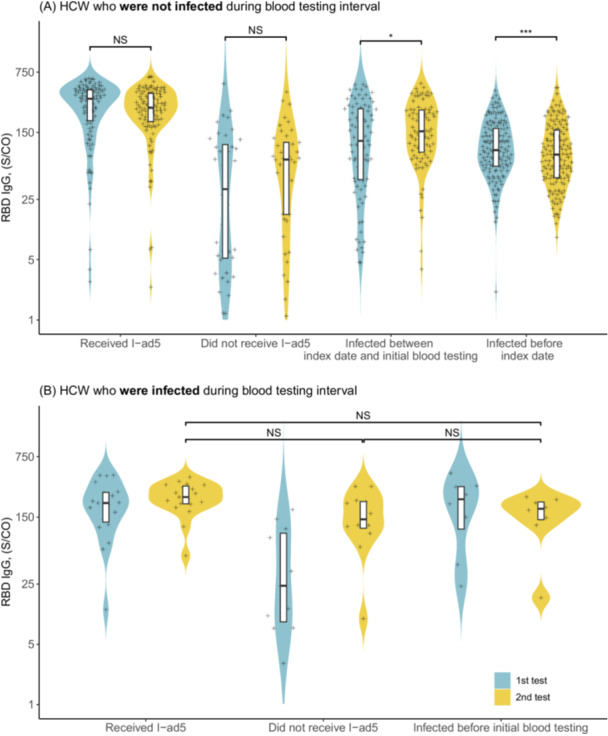

## 1 Background

The ongoing COVID‐19 epidemics are now mostly driven by the SARS‐CoV‐2 Omicron subvariants, with the Omicron BA. 2.86 and JN.1, which are considered as the variants of interest by the World Health Organization (WHO), circulating across the globe since November 2023 [1]. Data have shown that BA.2.86 is less immune‐invasive compared to the previous dominant strain, XBB, but is similar to the BA.2 and BA.4/5 strains antigenically [2]. Having caused multiple outbreaks globally since mid‐2022, the Omicron BA.5 variants were designated as variants of concern by the WHO and surpassed the BA.2 and BA.4 to become the dominant global strain until early 2023 [3].

Since October 2022, an inhalable COVID‐19 vaccine based on adenovirus type 5 vector (inhaled Ad5‐nCoV [I‐ad5]) has been granted for use in many regions, including China [4], providing a new method for delivering the vaccine. The I‐ad5 vaccine, developed by CanSinoBIO and the Military Academy of Medical Sciences, utilizes a recombinant adenovirus type 5 vector carrying the S gene of the ancestral SARS‐CoV‐2 strain, which encodes the full‐length spike (S) protein of the virus. This S protein stimulates the human immune system to generate both neutralizing antibodies and cellular immune responses against SARS‐CoV‐2. In contrast to the traditional intramuscularly administered vaccine, the I‐ad5 could be delivered and stored with minimal resources. Besides, I‐ad5 could provoke immune responses in the mucosal site, where the pathogens would attach, to potentially limit the viral shedding and transmission [5]. One previous study has indicated a moderate vaccine effectiveness provided by I‐ad5 as a second booster dose against the Omicron BA.5 variants. On the other hand, it is also crucial to assess the temporal change in the immune response induced by I‐ad5 to identify the waning in immunity and to potentially inform the vaccination strategy, given a rapid waning of the immunity provided by vaccination or infection against the Omicron variants [6, 7].

So far, only a few clinical trials have shown the immunogenicity of the I‐ad5 among the healthy general population [8, 9], and there is a lack of real‐world data on the immune profile among high‐risk groups, including healthcare workers (HCW), who were on the frontline facing SARS‐CoV‐2 infections, particularly during an epidemic. In China, the largest outbreak hitherto, which occurred in late 2022, was initiated by the Omicron BA.5 variants [10]. This outbreak offered an opportunity to conduct an observational study of the immune profile of I‐ad5 among HCW.

## 2 Methods

Urumqi, a Northern city of the Xinjiang Uygur Autonomous Region, China, saw a COVID‐19 outbreak seeded by the Omicron BA.5 variants from August to December 2022, when the “zero COVID‐19” policy was imposed [11]. A cohort comprised of all HCW from the Sixth Affiliated Hospital of Xinjiang Medical University was recruited on November 22 and 23. Participants were asked to fill out questionnaires to provide information on demographic (age and sex), baseline health status (body mass index [BMI], number of comorbidities, and smoking status), occupation, history of previous SARS‐CoV‐2 infection (individuals who had prior infection (Omicron BA.5) had three intramuscularly injected vaccine before the infection date), vaccination status (intramuscularly injected vaccination date and dosage) and the willingness of receiving serial blood tests. We excluded participants without a known history of SARS‐CoV‐2 infection, those who declined serial blood tests, and those who withdrew from the study. All participants with questionnaire interviews or blood sample collection in this study have signed paper‐based information consent forms.

The I‐ad5 was administered intranasally to enrolled individuals without a history of SARS‐CoV‐2 infection between November 22 and 23. In total, 3 μL of venous blood were collected from the arm two times on December 28, 2022, and on March 1, 2023 (blood testing interval), at the laboratory‐test center of the hospital, after which an IgG diagnostic test was performed immediately. The magnetic particle chemiluminescence method was performed to detect IgG antibodies against the receptor‐binding domain of the SARS‐CoV‐2 spike protein (anti S‐RBD, ancestral strain RBD). The IgG test kit used in this study was the Diagnostic Kit for Novel Coronavirus (2019‐nCoV) IgG Antibody (Magnetic Particle CLIA) (100 tests/kit), developed by Autobio Diagnostics Co. Ltd. (Zhengzhou, China). The relative light unit (RLU) could be read from the IgG test kit after a series of automatically performed testing tasks, and the obtained RLU was converted to titer units of S/CO. The follow‐up started from November 26 (the index date) till the date of the second blood sample test. The infection status was updated every 2 days by the reverse transcription polymerase chain reaction test (RT‐PCR) using the 2019‐nCoV Nucleic Acid Detection Kit (Fluorescent PCR Method) (32 tests/kit) manufactured by Zhong Yuan Hui Ji Biological Technology Co. Ltd. (Chongqing, China) during the follow‐up period.

Given that a subset of participants were infected during the blood testing interval, we classified the eligible individuals into two broad groups. Within each broad group, we further divided participants into seven subgroups according to the type of the last exposure type (i.e., vaccination or infection) before initial blood testing. Specifically, individuals who were not infected during the testing interval were categorized into four subgroups based on the last exposure status before the initial blood testing (received I‐ad5 group, not received I‐ad5 group, infected before index date, and infected between index date and initial blood testing). Likewise, individuals who were infected during the testing interval were assigned to three subgroups, namely, those who received I‐ad, did not receive I‐ad5, and those with a prior infection before initial blood testing. Distribution of IgG levels was plotted for each patient subgroup, and univariate comparisons of IgG levels were applied between subgroups by using the nonparametric test. We used linear mixed‐effect models to examine changes in IgG antibody levels between two tests among participants who were not infected during the interval, treating participants as a random effect. Age groups (18–30, 31–45, and > 45 years), sex, BMI group, smoking status, number of comorbidities, occupation group, and time lag to the first blood test were considered as confounding variables and were included in the mixed‐effect model as fixed effects. The effect size was computed as the weekly percentage change of antibody level between two tests. We did not include the vaccination (intramuscular injected vaccine) status as a confounding variable, as most of the HCW have received three doses of vaccine before follow‐up. The normality assumption was assessed visually using quantile–quantile (Q–Q) plots of residuals from the multivariate mixed‐effects models. We considered 0.05 as the level of statistical significance.

All statistical analyses were performed by using R statistical software (version 4.2.2) (R Program for Statistical Computing). The linear mixed‐effects models were fitted using the R package *lme4*, and figures were generated using *ggplot2*.

## 3 Results

**Figure 1:**
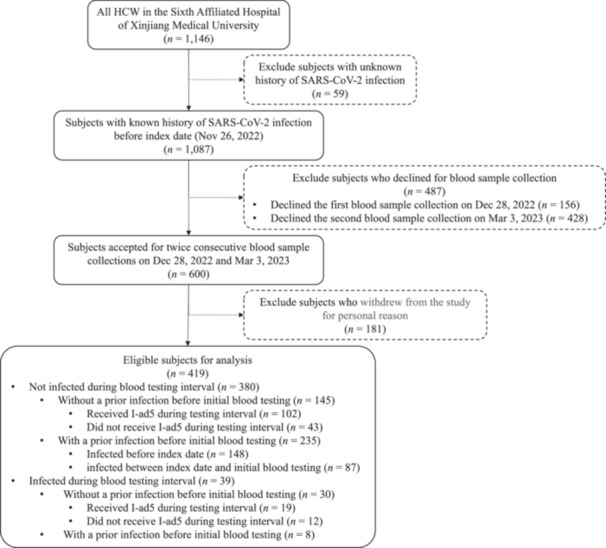
Flow chart of recruitment of study participants.

A total of 1146 HCW were recruited for screening, among which 419 HCW who had known infection status and accepted blood sample collection were included for further analysis (Figure 1). Of the 419 HCW, the majority of them were female (71.8%), doctors or nurses (73.2%), nonsmokers (87.6%), had received 3 doses of intramuscularly injected inactivated vaccine before follow‐up (93.8%), and had no history of comorbidities (94.3%) (Tables S1 and S2).

**Figure 2:**
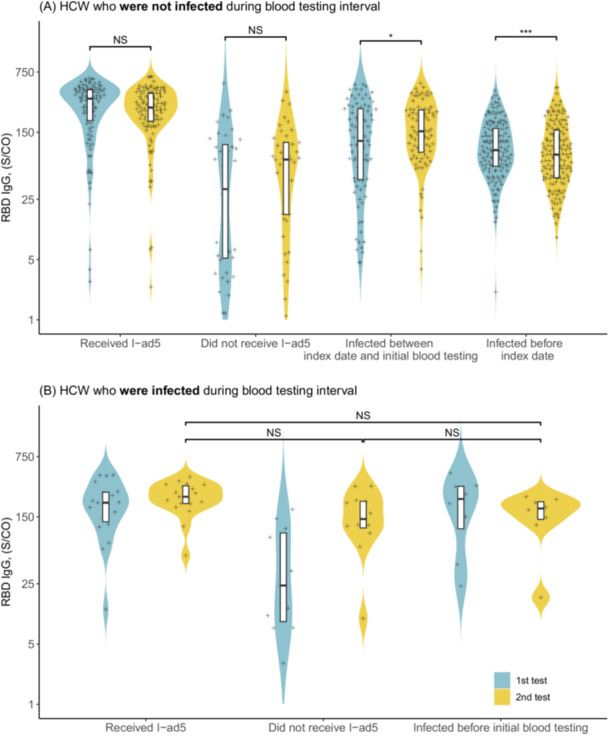
The distribution of anti‐RBD IgG antibody level (S/CO) from two blood tests among HCW who were infected by the Omicron BA.5 variants or received the I‐ad5. (A) The anti‐RBD IgG antibody level among HCW who were not infected during the blood testing interval. Wilcoxon signed‐ranks test, *p < 0.05, ***p < 0.001. (B) The anti‐RBD IgG antibody level among HCW who were infected during the blood testing interval. Wilcoxon rank sum test, *p*‐value was adjusted by Holm's approach. The boxplots in (A) and (B) showed the median, 25th and 75th percentiles of the observed distributions. The *y*‐axis was log‐transformed though the values on the ticks remained untransformed.

We investigated the temporal change in the immunity against SARS‐CoV‐2 among 380 HCW who were not infected during the blood testing interval (December 28, 2022, and March 1, 2023). The time lags between the last exposure type (vaccination or infection) and first blood test differed substantially between subgroups, with a shorter lag for HCW who were infected between the index date and initial blood testing (median: 8 days) than for those who were infected before the index date (median: 75 days) (Table S1). Univariate analysis showed a significant decrease in the IgG level between two tests for HCW who were infected before the index date and a significant increase for HCW who were infected between the index date and initial blood testing (Figure 2A). After adjusting for potential confounding variables, we estimated the IgG level rose by 5.1% (95% CI [confidence interval]: 2.4, 7.9) per week within around 2 months after Omicron BA.5 infection. No significant change in IgG level between the two tests was found for HCW without infection during the blood testing interval who received the I‐ad5. Approximately two and a half months after the BA.5 infection (for HCW infected before the index date), the difference in IgG level between the two tests was not significant (Table 1). Furthermore, we found the IgG level for HCW who did not receive the inhaled vaccine significantly increased by 7.7% (95% CI: 2.1, 13.6) per week between the two blood tests (Table 1). In addition, HCWs who were infected by the BA.5 after the first blood testing had similar intervals between infection and the second test (around 2 months; Table S1), and no significant difference in IgG was observed in group‐wise comparisons (Figure 2B). Notably, the CI range was significantly wider at the initial test (Test 1) compared to the second test (Test 2). The broader CI at Test 1 in infected HCW (Figure 2B) likely reflects heterogeneous immune responses (e.g., variable time since infection or preexisting immunity), which drove divergent antibody trajectories early postexposure. By Test 2 (~ 2 months later), antibody levels stabilized across subgroups (e.g., rising in new infections vs. plateauing in reinfections), narrowing the CI.

**Table 1:** Results of multivariate linear mixed‐effect models for HCW who were not infected by BA.5 during blood testing interval (*n* = 380).

Variable of interest	Effect size^a^ (95% CI)	*p*
Received I‐ad5 (*n* = 102)	−0.5% (−1.6, 0.7)	0.418
Did not receive I‐ad5 (*n* = 43)	7.7% (2.1, 13.6)	0.009
Infected between the index date and initial blood testing (*n* = 87)	5.1% (2.4, 7.9)	< 0.001
Infected before index date (*n* = 148)	−1.1% (−2.7, 0.5)	0.168

^a^ The effect sizes were weekly percentage change of IgG levels, adjusting for age, sex, BMI, ethnicity, occupation, smoking status, and the time lag to the first blood sample test as fixed effects in the linear mixed‐effect models.

## 4 Discussion

The anti‐RBD antibodies are correlated with the neutralizing antibodies, both of which are protective correlates of SARS‐CoV‐2 infection [12, 13]. During the study period (December 2022–March 2023), national surveillance data indicated that the BA.5.2 sublineage dominated SARS‐CoV‐2 transmission in China, which aligns with our focus on BA.5‐induced immune responses. Our findings suggested that the anti‐RBD IgG conferred by Omicron BA.5 infection increased rapidly within 2 months after infection and may remain unchanged during the third and fourth months after infection. Previous studies reported a substantial increase in the IgG level 100 days after infection by the historical strains [14] and 5–7 weeks after the Omicron breakthrough infection (without prior infection) [15].

As a novel vaccination strategy, the I‐ad5 used as a booster regimen (after two doses of inactivated intramuscular vaccination) could induce greater neutralizing antibodies than three doses of inactivated intramuscular vaccine [16]. We found that the IgG antibody induced by a booster I‐ad5 (the fourth dose after three doses of inactivated intramuscular vaccine) did not drop significantly in short term (the third month since vaccination) after 1 month of vaccination, which is consistent with a previous clinical trial studying the immunogenicity of the I‐ad5, showing that there was no decrease in the RBD‐binding antibodies against BA.4/5 between the first and the third months since the receipt of I‐ad5 as a heterologous booster after two inactivated vaccine among healthy Chinese adults [9]. Similarly, one study of the duration of the neutralizing antibody against the BA.4/5 demonstrated that the seropositivity of healthy adults who received I‐ad5 as a heterologous booster persisted for 12 months [8]. The present findings were also partially corroborated with previous data for HCW who received three doses of mRNA vaccine, suggesting a slight decrease in IgG level 1 month after vaccination [17]. Therefore, booster doses of vaccine should be promoted among HCW against novel variants with similar virological and epidemiological characteristics to the BA.5.

Although we observed a significant increase in IgG antibody for HCW who never infected or received I‐ad5, the IgG level remained at a lower level even after a 2‐month increase (from a median of 21.6–39, with a weekly increasing rate of 7.7%). We speculated that such an increase in IgG may be due to the frequent interaction with the SARS‐CoV‐2 fomite in the hospital. Additionally, the IgG level differed insignificantly among newly Omicron BA.5 infected HCW during follow‐up, who had different levels of immunity before the infection (vaccination or infection before follow‐up). This implies the IgG antibody level may mostly depend on the most recent exposure. The limitations of the study included a relatively small sample size and the absence of serologic assays measuring the neutralizing antibodies and anti‐RBD antibodies against the SARS‐CoV‐2 strains. Future studies should investigate the long‐term profile of RBD IgG antibody after vaccination or infection and examine whether the findings in this work could be generalized to other types of vaccines and emerging SARS‐CoV‐2 variants.

Although our analysis found no significant association between BMI and infection, obesity may reduce vaccine efficacy or accelerate antibody waning via immunometabolic disturbances. Future studies should integrate metabolic profiling (e.g., inflammatory markers, adipokine levels) with longitudinal immune monitoring to clarify these relationships in populations with heterogeneous metabolic health.

In conclusion, our study indicated that the initial increase in the anti‐RBD IgG elicited by the Omicron BA.5 variants remained at the third and fourth months after infection and that the antibodies induced by the I‐ad5 as a booster dose could persist in the third month after 1 month of vaccination, adding to the data on the immune profile among HCW who received the I‐ad5. These findings suggested that I‐ad5 may provide modest protection against Omicron BA.5 infection in HCW population.

## Author Contributions

**Zhaohui Luo:** data curation, formal analysis, investigation, writing – original draft. **Ting Zeng:** funding acquisition, investigation, writing – original draft. **Shi Zhao:** conceptualization, methodology, writing – review and editing. **Jing Liang:** data curation, resources, writing – review and editing. **Shengzhi Sun:** writing – review and editing. **Yongkang Ni:** writing – review and editing. **Chunyan Yan:** data curation, writing – review and editing. **Liang Yin:** data curation, writing – review and editing. **Lan Wang:** data curation, writing – review and editing. **Kai Wang:** funding acquisition, project administration, supervision, writing – review and editing. **Zihao Guo:** conceptualization, formal analysis, methodology, software, supervision, writing – original draft. All authors critically read the manuscript and gave final approval for publication.

## Ethics Statement

This study was approved by the Institutional Ethics Committee of Xinjiang Medical University (IRB No.: XJYKDXR20221001001).

## Consent

All participants with questionnaire interview or blood sample collection in this study have signed paper‐based information consent forms. Medical records were kept confidential in full at the Sixth Affiliated Hospital of Xinjiang Medical University, and the personal identity of the subjects was not and will not be disclosed in any report on the results of this study. Patients or the public were not involved in the design, conduct, reporting, or dissemination plans of this study.

## Conflicts of Interest

The authors declare no conflicts of interest.

## Supporting information


**Table S1:** Demographic and clinical characteristics of HCW who were not infected by BA.5 during blood testing interval (*n* = 380). **Table S2:** Demographic and clinical characteristics of HCW who were infected by BA.5 during blood testing interval (*n* = 39).

## Data Availability

The original database containing confidential patient information cannot be made publicly available. The anonymized data used in this study were available based on a reasonable request to the corresponding authors.
