# Supplementary material for: Short‐Term Change in IgG Antibody Elicited by Omicron BA.5 Infection and Inhaled Ad5‐nCoV Vaccine Among Healthcare Workers
Source: Immun Inflamm Dis. 2026 Jan 19;14(1):e70332. doi: 10.1002/iid3.70332 (PMC12815692; doi:10.1002/iid3.70332)
Supplement: Supplementary file 1 — Table S1: Demographic and clinical characteristics of HCW who were not infected by BA.5 during blood testing interval (n = 380). Table S2: Demographic and clinical characteristics of HCW who were infected by BA.5 during blood testing interval (n = 39). [file IID3-14-e70332-s001.docx]

**Supplementary materials**

**Table S1.** Demographic and clinical characteristics of HCW who were not infected by BA.5 during blood testing interval (*n* = 380).

| **Characteristics** | **Received I-ad5**  **(*n* = 102)** | **Did not receive I-ad5 (*n* = 43)** | **Infected between index date and initial blood testing**  **(*n* = 87)** | **Infected before index date (*n* = 148)** |
| --- | --- | --- | --- | --- |
| Sex | | | | |
| Male | 41 (40.1%) | 12 (27.9%) | 18 (20.7%) | 36 (24.3%) |
| Female | 61 (59.9%) | 31 (72.1%) | 69 (79.3%) | 112 (76.7%) |
| Age (years) | | | | |
| Median (IQR) | 35 (28, 44) | 38 (30, 51) | 33 (30, 42) | 35 (29, 42) |
| 18-30 | 34 (33.3%) | 11 (25.6%) | 31 (35.7%) | 47 (31.8%) |
| 31-45 | 45 (44.1%) | 15 (34.9%) | 41 (47.1%) | 79 (53.3%) |
| >45 | 23 (22.6%) | 17 (39.5%) | 15 (17.2%) | 22 (14.9%) |
| BMI group | | | | |
| Underweight (<18.5) | 8 (7.8%) | 0 (0.0%) | 8 (9.2%) | 5 (3.4%) |
| Normal (18.5-24) | 51 (50.0%) | 21 (48.9%) | 33 (37.9%) | 67 (45.3%) |
| Overweight (25-29) | 33 (32.4%) | 17 (39.5%) | 29 (33.3%) | 59 (39.9%) |
| Obese (≥30) | 10 (9.8%) | 5 (11.6%) | 17 (19.6%) | 17 (11.4%) |
| Ethnicity | | | | |
| Han | 89 (87.3%) | 35 (81.4%) | 59 (67.8%) | 110 (74.3%) |
| Others | 13 (12.7%) | 8 (8.6%) | 28 (32.2%) | 38 (25.7%) |
| Occupation | | | | |
| Doctor or nurse | 71 (69.6%) | 28 (65.1%) | 71 (81.6%) | 107 (72.3%) |
| Others | 31 (30.4%) | 15 (34.9%) | 16 (18.4%) | 41 (27.7%) |
| Smoking status | | | | |
| Smoker | 19 (18.6%) | 5 (11.6%) | 7 (8.0%) | 16 (10.8%) |
| Non-smoker | 83 (81.4%) | 38 (88.4%) | 80 (92.0%) | 132 (89.2%) |
| Vaccine dosage before follow-up^a^ | | | | |
| 0-2 | 3 (2.9%) | 6 (14.0%) | 3 (3.4%) | 11 (7.4%) |
| 3 | 99 (97.1%) | 37 (86.0%) | 84 (96.6%) | 137 (92.6%) |
| Number of comorbidities |  |  |  |  |
| 0 | 94 (92.2%) | 37 (86.0%) | 86 (98.9%) | 142 (95.9%) |
| 1 | 8 (7.8%) | 4 (9.3%) | 1 (1.1%) | 5 (3.4%) |
| ≥2 | 0 (0.0%) | 2 (4.7%) | 0 (0.0%) | 1 (0.7%) |
| IgG level (S/CO), median (IQR) | | | | |
| First blood test (Dec 28, 2022) | 369.0 (205.0, 469.0) | 21.6 (3.5, 103.0) | 108.0 (40.7, 280.0) | 92.1 (58.1, 166.0) |
| Second blood test (Mar 3, 2023) | 291.0 (201.0, 426.0) | 72.0 (14.4, 113.0) | 154.0 (85.1, 271.0) | 81.5 (44.3, 159.0) |
| Lag to the first blood test^b^ (days), median (IQR) | 36 (36, 36) | 407 (376, 426) | 8 (6, 11) | 75 (66, 81) |

^a^The inactivated vaccine before follow-up was administered intramuscularly.

^b^Received I-ad5 group: the time interval between the date of receiving I-ad5 and initial blood testing; Did not receive I-ad5 group: the time interval between the date of receiving the last intramuscularly injected vaccine and initial blood testing; Infected between index date and initial blood testing/before index date: the time interval between the date of infection and initial blood testing.

**Table S2**. Demographic and clinical characteristics of HCW who were infected by BA.5 during blood testing interval (*n* = 39).

| **Characteristics** | **Received I-ad5**  **(*n* = 19)** | **Did not receive I-ad5 (*n* = 12)** | **With a prior infection before initial blood testing** **(*n* = 8)** |
| --- | --- | --- | --- |
| Sex | | | |
| Male | 4 (21.2%) | 4 (33.3%) | 3 (37.5%) |
| Female | 15 (78.8%) | 8 (66.7%) | 5 (62.5%) |
| Age (years) | | | |
| Median (IQR) | 31 (29, 41) | 39 (35, 51) | 35 (31, 42) |
| 18-30 | 8 (42.0%) | 3 (25.0%) | 2 (25.0%) |
| 31-45 | 7 (36.8%) | 4 (33.3%) | 4 (50.0%) |
| >45 | 4 (21.2%) | 5 (41.7%) | 2 (25.0%) |
| BMI group | | | |
| Underweight (<18.5) | 4 (21.2%) | 0 (0.0%) | 0 (0.0%) |
| Normal (18.5-24) | 9 (47.2%) | 4 (33.3%) | 3 (37.5%) |
| Overweight (25-29) | 5 (26.3%) | 6 (50.0%) | 4 (50.0%) |
| Obese (≥30) | 1 (5.3%) | 2 (16.7%) | 1 (12.5%) |
| Ethnicity | | | |
| Han | 17 (89.4%) | 8 (66.7%) | 3 (37.5%) |
| Others | 2 (10.6%) | 4 (33.3%) | 5 (62.5%) |
| Occupation | | | |
| Doctor or nurse | 17 (69.6%) | 7 (65.1%) | 6 (75.0%) |
| Others | 2 (30.4%) | 5 (34.9%) | 2 (25.0%) |
| Smoking status | | | |
| Smoker | 3 (15.8%) | 1 (8.3%) | 1 (12.5%) |
| Non-smoker | 16 (84.2%) | 11 (91.7%) | 7 (87.5%) |
| Vaccine dosage before follow-up^a^ | | | |
| 0-2 | 1 (5.3%) | 2 (16.7%) | 0 (0.0%) |
| 3 | 18 (94.7%) | 10 (83.3%) | 8 (100.0%) |
| Number of comorbidities | | | |
| 0 | 18 (94.7%) | 11 (91.7%) | 7 (87.5%) |
| 1 | 1 (5.3%) | 1 (8.3%) | 1 (12.5%) |
| ≥2 | 0 (0.0%) | 0 (0.0%) | 0 (0.0%) |
| IgG level (S/CO), median (IQR) | | | |
| First blood test (Dec 28, 2022) | 218.0 (132.0, 290.0) | 18.4 (7.8, 92.3) | 243.0 (123.0, 338.0) |
| Second blood test (Mar 3, 2023) | 256.0 (213.0, 343.0) | 143.0 (111.0, 227.0) | 187.0 (140.0, 224.0) |
| Lag to the second blood sample test^b^ (days), median (IQR) | 59 (54, 60) | 56 (50, 60) | 44 (8, 73) |

^a^The inactivated vaccine before the follow-up was administered intramuscularly.

^b^With/without I-ad5 received group: the time interval between the date of infection and second blood testing; Reinfected group: the time interval between the date for the last infection and second blood testing
